# Case Report: Inotersen reintroduction after thrombocytopenia in hereditary transthyretin amyloidosis: a case series

**DOI:** 10.3389/fphar.2026.1906711

**Published:** 2026-08-28

**Authors:** Cesar A. Crespi, Gisela Zanga, Maria Florencia Ardanaz, Marcela Pia Chinigo, Graciana Bianco, Andrea Del Mar Ibañez Aldecoa

**Affiliations:** 1 Centro de Referencia en Enfermedades Raras y de Dificultoso Diagnóstico (CERyD), Hospital Interzonal Especializado de Agudos y Crónicos San Juan de Dios, La Plata, Argentina; 2 Servicio de Neurología, Hospital Cesar Milstein, Buenos Aires, Argentina; 3 Servicio de Alergia, Hospital Interzonal Especializado de Agudos y Crónicos San Juan de Dios, La Plata, Argentina; 4 Servicio de Farmacia, Hospital Interzonal Especializado de Agudos y Crónicos San Juan de Dios, La Plata, Argentina

**Keywords:** ATTRv, case series, hereditary transthyretin amyloidosis, inotersen, real-world evidence, rechallenge, reintroduction, thrombocytopenia

## Abstract

**Background:**

ATTRv amyloidosis is a progressive multisystem disease caused by pathogenic variants in the transthyretin (TTR) gene. Inotersen is approved for the treatment of polyneuropathy in adults with hereditary transthyretin mediated amyloidosis, but thrombocytopenia is a clinically relevant adverse event that may require treatment interruption or discontinuation. Evidence guiding reintroduction of inotersen after thrombocytopenia is limited.

**Case Presentation:**

We retrospectively reviewed two men with genetically confirmed ATTRv treated at two centers in Argentina who developed treatment-emergent thrombocytopenia during inotersen therapy. Patient 1 was a 71-year-old man with late-onset mixed neurologic and cardiac ATTRv due to TTR p.Val50Met who developed grade 2 thrombocytopenia after 22 weeks of treatment, with a platelet nadir of 58 × 10^9/L. Patient 2 was a 52-year-old man with early-stage neuropathic ATTRv due to TTR p.Val50Met who developed grade 4 thrombocytopenia after 65 weeks, with platelet counts below 20 × 10^9/L, and had recurrent thrombocytopenia during an initial reintroduction attempt.

**Intervention and Outcomes:**

After platelet recovery and exclusion of alternative causes of thrombocytopenia, both patients underwent a structured 7-day stepwise short-term reintroduction protocol under close hematologic monitoring. Following protocol completion, inotersen was resumed at 284 mg every 2 weeks. During follow-up, neither patient developed recurrent severe thrombocytopenia, and both maintained biochemical evidence of transthyretin suppression.

**Conclusion:**

In this small case series, inotersen reintroduction after thrombocytopenia was feasible in two ATTRv patients when implemented with a structured stepwise protocol and close platelet monitoring, but these findings are hypothesis-generating and not generalizable. One reintroduction occurred outside prescribing recommendations for severe thrombocytopenia and should not be interpreted as a recommended strategy.

## Introduction

Hereditary transthyretin amyloidosis (ATTRv) is a progressive inherited disorder caused by pathogenic variants in the *TTR* gene, resulting in misfolded transthyretin deposition in peripheral nerves, the heart, and other tissues. Clinical manifestations include progressive polyneuropathy, autonomic dysfunction, cardiomyopathy, and substantial functional impairment. Disease-modifying therapies that reduce hepatic transthyretin production have significantly changed the management of this condition (Adams et al., 2016).

Inotersen is an antisense oligonucleotide that reduces hepatic production of transthyretin (TTR). Although it is no longer marketed in the United States or Canada, it remains available in several other countries. Inotersen has been shown to slow neurologic progression in patients with ATTRv. However, thrombocytopenia is a recognized adverse event and may necessitate treatment interruption or permanent discontinuation. In the pivotal study by Benson et al. (2018), severe thrombocytopenia (i.e., very low platelet counts) was uncommon, occurring in approximately 3% of inotersen-treated patients. Current risk-mitigation strategies center on regular platelet monitoring with dose adjustment or discontinuation in response to clinically relevant declines. While this approach improves safety, it does not address a key practical question: whether—and how—inotersen can be reintroduced after thrombocytopenia in selected patients.

This question is particularly relevant in settings in which access to alternative disease-modifying therapies is delayed or restricted, especially in low- and middle-income countries, where therapeutic options may be limited by cost, reimbursement, or logistical barriers. In such circumstances, treatment discontinuation may result in clinical deterioration during the interval before another therapeutic option becomes available. Despite a comprehensive review of the available literature, we found no published cases addressing reintroduction of inotersen after thrombocytopenia.

We therefore report two cases of patients with ATTRv who developed thrombocytopenia during inotersen therapy and were subsequently managed with a structured stepwise reintroduction strategy under close hematologic surveillance.

## Objective

To describe the feasibility and short-term safety of a structured stepwise reintroduction protocol for inotersen in patients with ATTRv who developed treatment-emergent thrombocytopenia.

## Methods

This was a retrospective, observational case series conducted at two centers in Argentina.

We included patients with genetically confirmed ATTRv who: received treatment with inotersen, developed thrombocytopenia during therapy, discontinued treatment because of thrombocytopenia, and subsequently underwent structured reintroduction after platelet recovery.

Clinical records were reviewed to obtain demographic and clinical data, TTR genotype, disease phenotype, neurologic stage according to the Polyneuropathy Disability (PND) score, cardiac involvement, duration of inotersen exposure before thrombocytopenia, platelet nadir, thrombocytopenia severity according to Common Terminology Criteria for Adverse Events (CTCAE), treatment modifications, and follow-up outcomes. Alternative causes of thrombocytopenia were assessed clinically and excluded in both patients.

### Reintroduction protocol

After recovery of platelet counts, both patients underwent a structured stepwise short-term reintroduction protocol consisting of sequential daily administration of increasing inotersen doses over 7 days: 3, 6, 12, 24, 48, 93, and 98 mg. This schedule allowed completion of a cumulative therapeutic dose over 1 week. Patients were followed within a patient support program with more than 6 years of experience in inotersen management, which enabled close safety surveillance throughout treatment and reintroduction (Tomei et al., 2025). Platelet monitoring included dual assessment by both automated platelet count and peripheral blood smear review. No premedication was administered.

Platelet counts were reassessed at 7 and 14 days after protocol completion and then monitored regularly. In the absence of recurrent thrombocytopenia, inotersen was resumed at a reduced frequency of 284 mg every 14 days, with subsequent adjustment according to platelet stability and clinical evolution.

### Ethical considerations

The study was conducted in accordance with the Declaration of Helsinki. All patients provided informed consent for the academic use of their clinical data.

### Stepwise short-term reintroduction protocol (SSRP)

In cases in which the platelet count fell below 100 × 10^9/L and did not recover with standard management—defined as extending the dosing interval of inotersen and resuming weekly administration only after three consecutive platelet counts above 100 × 10^9/L—patients were referred to the Allergy Department for an inotersen reintroduction protocol.

**FIGURE 1:**
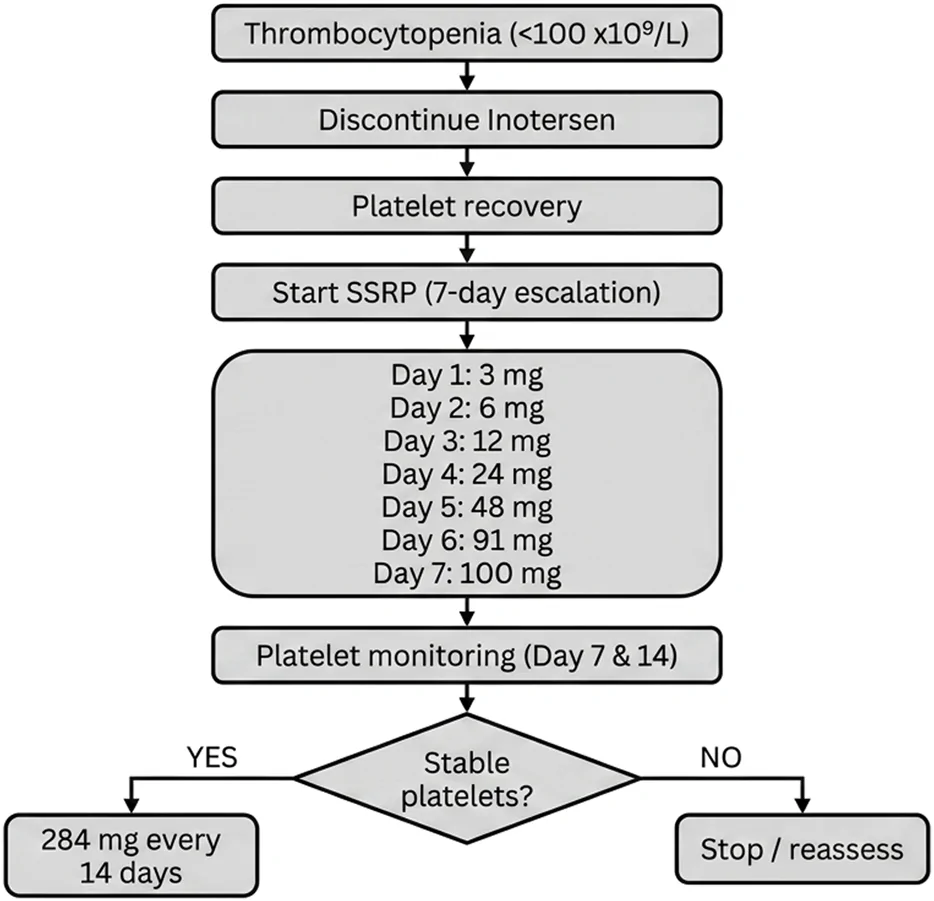
Stepwise short-time reintroduction protocol (SSRP) for reintroduction of inotersen following thrombocytopenia.

Reintroduction was performed in our patients with hereditary transthyretin amyloidosis using a modified protocol based on the case report by Isaac et al. (2023), who described hypersensitivity desensitization to the antisense oligonucleotide volanesorsen. Inotersen was prepared from a 284 mg/1.5 mL prefilled syringe under sterile conditions in a laminar flow hood and aseptically transferred into disposable sterile syringes containing the required volumes for each dose, which were then capped, labeled, and dispensed with storage instructions. The protocol consisted of seven consecutive days of graded re-exposure with escalating doses of 3, 6, 12, 24, 48, 91, and 100 mg. Dose fractionation was performed using insulin syringes (1 unit = 10 µL). Based on the nominal/approximate inotersen concentration in the prefilled syringe (≈0.2 mg/μL), the corresponding approximate injection volumes were 1.5, 3, 6, 12, 24, 48, and 50 units, respectively (Figure 1).

The protocol consisted of daily sequential administration of increasing doses over consecutive days, according to the following schedule:

- Day 1: 3 mg
- Day 2: 6 mg
- Day 3: 12 mg
- Day 4: 24 mg
- Day 5: 48 mg
- Day 6: 91 mg
- Day 7: 100 mg

This approach allowed completion of a cumulative full therapeutic dose within 1 week. No premedication was administered.

During graded re-exposure, platelet counts were monitored every 48 h during dose escalation, then weekly for 1 month, and subsequently every 2 weeks (ideally the day before dosing). Platelets were reassessed 7 and 14 days after protocol completion. If no recurrent thrombocytopenia occurred, treatment was resumed at a reduced frequency (284 mg every 14 days), with further adjustments guided by platelet stability.

Plasma transthyretin (TTR) levels were measured during follow-up, confirming sustained pharmacodynamic suppression under the modified dosing regimen.

The protocol was designed to enable controlled re-exposure while minimizing abrupt reintroduction and allowing early detection of hematologic changes.

## Case presentation–results

### Case 1

A 71-year-old man with genetically confirmed ATTRv due to *TTR* p.Val50Met presented with late-onset disease and a mixed phenotype. He had a 7-year history of sensory-motor and autonomic polyneuropathy, classified as PND stage II, together with cardiac involvement supported by Perugini grade 3 uptake on bone scintigraphy.

Because of ongoing disease progression, treatment with inotersen 284 mg weekly was initiated in December 2024. After 22 weeks of therapy, he developed thrombocytopenia with a platelet nadir of 58 × 10^9/L, consistent with CTCAE grade 2 toxicity. No bleeding manifestations were observed, and treatment was interrupted.

Following platelet recovery, a structured stepwise reintroduction protocol was initiated in July 2025 under close hematologic monitoring. After protocol completion, treatment was resumed at 284 mg every 2 weeks. During follow-up, no recurrent severe thrombocytopenia was observed (Table 1). No clinical dysautonomia was identified during follow-up. NT-proBNP remained within normal limits. No additional adverse events were reported, including no injection-site reactions and no systemic flu-like symptoms.

**TABLE 1:** Clinical characteristics and outcomes of patients undergoing inotersen reintroduction after thrombocytopenia.

Variable	Case 1	Case 2
Age/sex	71/M	52/M
BMI (kg/m^2^)	23	21
SCr mg/dl	0,51	0.75
TTR variant	p.Val50Met	p.Val50Met
PLT baseline	158	173
PLT nadir	58	18
Time to thrombocytopenia, wk	22	65
Time to SSRP, wk*	10	12 and 22
Mean PLT post-reintroduction	114	140
Recurrence	No	No
Follow-up, mo**	18 months	20 months
Inotersen interval dose	2 w	2 w
Prealbumin reduction***	13 mg/dL (<LLN)	10 mg/dL (<LLN)

BMI: body mass index, SCr: Serum creatinine, SSRP, stepwise short-term reintroduction protocol; PLT, platelet count; mo, months; wk, weeks; LLN, lower limit of normal.

* Time to reintroduction calculated from platelet recovery after treatment discontinuation.

** Follow-up is reported from inotersen initiation.

*** Reference range: 20–40 mg/dL.

### Case 2

A 52-year-old man with genetically confirmed ATTRv due to *TTR* p.Val50Met presented with early-stage neuropathic disease (PND stage I). He began treatment with inotersen 284 mg weekly in August 2022.

In November 2023, after 65 weeks of treatment, he developed severe thrombocytopenia with platelet counts below 20 × 10^9/L, corresponding to CTCAE grade 4 toxicity. He required hospitalization and corticosteroid therapy, and inotersen was discontinued. Meprednisone 60 mg/day was administered for 2 days, and platelet counts recovered within 72 h–83 × 10^9/L. At the platelet nadir, peripheral blood smear was reviewed repeatedly (daily during hospitalization), excluding pseudo thrombocytopenia. Viral studies were negative and there were no clinical signs of systemic infection. Liver disease/splenomegaly and bleeding manifestations were not present. Concomitant medications were limited to pregabalin and tramadol. Anti-platelet antibody testing was not available. Given an otherwise unremarkable blood count and rapid platelet recovery after corticosteroids, bone marrow studies were deferred.

The first reintroduction attempt under close monitoring was followed by recurrent thrombocytopenia, with platelet counting below 80 × 10^9/L. Subsequently, the patient underwent a structured stepwise reintroduction protocol on two separate occasions in April and August 2024. This strategy ultimately allowed treatment reintroduction and maintenance on inotersen 284 mg every 2 weeks. During follow-up, he remained clinically and biochemically stable, without further severe hematologic adverse events. No clinical dysautonomia was identified during follow-up. NT-proBNP remained within normal limits. No additional adverse events were reported, including no injection-site reactions and no systemic flu-like symptoms.

Both patients were successfully re-exposed to inotersen after prior treatment-emergent thrombocytopenia. Following implementation of the structured reintroduction protocol and continuation at a reduced dosing frequency, neither patient experienced recurrent severe thrombocytopenia during follow-up (Table 1).

## Discussion

This two-patient case series describes the reintroduction of inotersen after clinically significant thrombocytopenia in patients with ATTRv. In both cases, treatment was resumed after platelet recovery using a structured stepwise protocol and continued at a reduced dosing frequency, without recurrence of severe thrombocytopenia during follow-up.

The main contribution of this report is practical rather than mechanistic. Current safety recommendations emphasize early recognition of thrombocytopenia and prompt treatment interruption, but there is little published evidence to guide whether reintroduction can be attempted safely in selected patients. Our cases suggest that, when therapeutic alternatives are limited and the expected clinical benefit remains meaningful, reintroduction may be feasible under intensive hematologic monitoring.

The broader literature on drug-induced thrombocytopenia provides a useful framework for interpreting our findings. Prior reviews have emphasized that clinically significant thrombocytopenia may result from either immune-mediated peripheral platelet destruction or non-immune mechanisms, and that diagnosis relies largely on the temporal association with drug exposure, recovery after withdrawal, and exclusion of alternative causes (Aster and Bougie, 2007; Arnold et al., 2013; Kuter, 2015). In this setting, prompt discontinuation of the suspected agent remains the cornerstone of management, and re-exposure is generally avoided when safer alternatives are available (Aster and Bougie, 2007; Arnold et al., 2013; Kuter, 2015).

Literature on drug hypersensitivity and graded re-exposure in oncology and other chronic diseases suggests that, in selected circumstances, carefully supervised stepwise re-exposure may enable continuation of an otherwise important therapy (Castells, 2017; Brennan et al., 2009). Although this experience cannot be directly extrapolated to inotersen-associated thrombocytopenia, it provides a conceptual rationale for the plausibility of a structured reintroduction approach when therapeutic options are limited. Accordingly, our observations should be interpreted cautiously: rather than demonstrating a defined tolerance mechanism, these cases suggest that individualized, closely monitored reintroduction may be feasible in exceptional circumstances. While the schedule was informed by graded re-exposure approaches described for other antisense therapies, we do not imply an IgE-mediated desensitization mechanism; we therefore refer to the intervention as controlled, dose-fractionated re-exposure with close platelet monitoring.

These findings should be interpreted with substantial caution. First, the sample size is extremely small, and no mechanistic studies were performed; therefore, our data do not support conclusions regarding immune tolerance, desensitization biology, or the pathophysiology of inotersen-associated thrombocytopenia. Unlike Patient 1, Patient 2 experienced a nadir platelet count <25 × 10^9/L, a scenario in which prescribing information discourages re-initiation. The decision to attempt a controlled, fractionated re-exposure was individualized, based on clinical severity, lack of timely locally available alternatives at that time, patient preference after counseling, and documented informed consent.

For these reasons, the present findings should not be interpreted as evidence that reintroduction is broadly safe. Rather, they suggest that a carefully selected, individualized, and closely monitored approach may be considered in exceptional circumstances. This issue may be especially relevant in low- and middle-income settings, where access to alternative therapies (like vutisiran and enplotersen) may be delayed by cost, regulatory approval, or logistical barriers. In such contexts, treatment interruption may result not only in clinical deterioration but also, in some cases, in progression to a stage at which patients may no longer be eligible for certain disease-modifying therapies. Further evidence from larger multicenter cohorts or registry-based analyses is needed to better characterize patient selection, timing, monitoring strategies, and outcomes after inotersen reintroduction. Other known inotersen adverse events were monitored in accordance with the prescribing information, and no additional adverse events have been observed to date.

## Conclusion

In this small case series, inotersen reintroduction after thrombocytopenia was feasible in two ATTRv patients when implemented via a structured, stepwise approach with close platelet monitoring. These findings are hypothesis-generating and not generalizable.

Notably, one reintroduction occurred outside prescribing recommendations for severe thrombocytopenia and should not be interpreted as a recommended strategy. In selected patients with limited alternatives, reintroduction may be considered only on an individualized risk–benefit basis with intensive monitoring.

## Data Availability

The original contributions presented in the study are included in the article/supplementary material, further inquiries can be directed to the corresponding author.
